# Benchmarking commercial large language models for gene–disease–phenotype extraction from full‐text human genetics literature

**DOI:** 10.1002/qub2.70050

**Published:** 2026-08-17

**Authors:** Danqing Yin, Matthew Ka Siu Leung, Darren Wan Ho Pun, Fiona Haixin Chen, Julie Yujin Kwon, Xinyi Lin, Joshua W. K. Ho

**Affiliations:** ^1^ School of Biomedical Sciences Li Ka Shing Faculty of Medicine The University of Hong Kong Hong Kong China; ^2^ Laboratory of Data Discovery for Health Hong Kong Science Park Hong Kong China; ^3^ ESF South Island School Hong Kong China

**Keywords:** biomedical curation, CHDgene, Claude‐Opus 4, congenital heart disease, DeepSeek‐R1, evidence synthesis, gene annotation, generative artificial intelligence, GPT‐4o, LLM

## Abstract

Manual curation of gene–disease–phenotype relationships from the human genetics literature is a persistent bottleneck for maintaining its bioinformatics databases. Whereas large language models (LLMs) offer a promising alternative, there is currently no systematic benchmark that evaluates whether state‐of‐the‐art commercial LLMs can perform this task reliably on the full‐text articles. To address this gap, we introduce a standardized benchmark comprising 406 full‐text articles covering 180 congenital heart disease‐associated genes, and a multi‐dimensional evaluation framework that incorporates fuzzy matching to account for synonyms and partial matches. We benchmarked seven state‐of‐the‐art LLMs, GPT‐4o, Claude‐Opus‐4, DeepSeek‐R1, Grok‐4, Qwen‐3.5, Gemini‐2.5 (Pro), and GPT‐5 on the extraction of structured gene, disease, and phenotype fields. The top‐performing model, Grok‐4, achieved 97.6% overall accuracy, whereas the lowest‐performing model reached approximately 88%, still surpassing many prior benchmarks employing zero‐shot or *n*‐shot prompting in biomedical relation extraction (RE) tasks. Our results provide a rigorous characterization of current LLMs capabilities and limitations. This paper contains two components. First, we conducted a human genetics field benchmark study on LLMs against a curated database. Second we developed the evaluation framework for this task. The benchmark dataset, evaluation framework, and model benchmarking outputs are made available online to support future studies in a reproducible manner.

## 1 INTRODUCTION

The growing pace of human disease genetic research has resulted in an unprecedented volume of scientific literature, the majority of it containing essential information about gene–disease associations, phenotypes, variants, and underlying pathological mechanisms.

Several recent studies have evaluated the use of generative artificial intelligence (AI) systems to extract biomedical relation extraction (RE) tasks. Although previous study [1] does not directly investigate the use of large language model (LLM) in extracting gene–disease–phenotype relationship, it applies RE on datasets, for example, ChemProt and DDI2013, which involves identifying relationships between protein‐protein and drug–drug interactions with the use of F1 scores for RE to provide benchmark for comparison with zero‐shot, few‐shot, fine‐tuning performances of GPT‐3.5, GPT‐4, and LLaMA 2 on 12 biomedical natural language processing (BioNLP) datasets. Another study [2] also investigated the use of ChatGPT on a broad set of BioNLP tasks, including RE by evaluating GPT‐3.5’s performance in extracting ChemProt, drug–drug interaction (DDI) and gene–disease associations database information using the biomedical language understanding and reasoning benchmark framework. Such a benchmarking framework can be adapted or extended for gene–disease–phenotype relationship extraction. Another pilot study [3] also evaluated multiple large language models (LLMs) in six BioNLP tasks including RE on 26 biomedical datasets including BC5CDR, KD‐drug target interaction, and DDI with BC5CDR being closely similar to gene–disease–phenotype extraction. In addition, LLM‐based open relation extraction is a framework introduced [4] for producing knowledge graphs of disease‐gene relationships mined from PubMed abstracts with 90% mean average precision. A team further assessed the use of GPT‐3.5 and GPT‐4 in extracting genetic and genomic data from full‐text journal articles [5], even though the tested literature is on wheat and barley genetics, the methodology applies to extracting gene‐phenotype and disease associations from unstructured scientific literature. What’s more, an automation framework has been proposed [6] for extracting disease–gene associations from literature which uses hit ratio to measure how well the framework ranks known disease‐gene associations. However, these evaluations primarily focus on structured benchmark datasets or short abstracts rather than full‐length human disease genetics literature. After all, in the field of human genetics, extraction of human gene–disease–phenotype information depends heavily on expert‐driven, manual extraction from the scientific paper, often in unstructured text. This is often a labor‐intensive and time‐consuming process that does not keep pace with the rate of human disease genetic discovery [7, 8].

Automation of the information extraction process using AI methods remains an important objective. Traditional text mining in biomedical research mostly relied on classical natural language processing methods [9]. These systems employed patterns, dependency parsing, and classifiers to extract genetic associations from abstracts and titles in literature repositories such as PubMed Central. Although reliable, interpretable, and causally aware AI agents are yet to be developed to accelerate biomedical knowledge discovery [4, 10]. Traditional BioNLP models are limited by their sentence‐level scope, reliance on annotated corpus, and inability to reason over full documents or multi‐hop relationships [11, 12]. Subsequent advances in BioBERT—a fine‐tuned large pretrained language model on biomedical corpora [8] has improved particularly on named entity recognition (NER) and RE and full document processing.

Recent emerging techniques in LLMs such as GPT‐3.5, GPT‐4, and BioGPT have opened new possibilities for zero‐ and few‐shot learning in biomedical information extraction [13]. These models demonstrate superficial fluency and task generalization, including for entity and RE without task‐specific training. Several studies have revealed the potentials of LLMs for tasks such as drug‐gene‐cancer triad extraction [14], gene‐trait retrieval from plant literature [5], structured evidence extraction from full‐text papers [4], and gene function extraction from full‐text papers [15] as well as genetic variant prediction from unstructured text [10]. However, most of these approaches rely on retrieval‐augmented generation (RAG), model pretraining, task‐specific fine‐tuning, or external confidence scoring pipelines, all of which involve engineering complexity, computational cost, and risks of hallucination. Despite the abovementioned technical challenges, human genetics literature itself is in nature highly sophisticated to parse. Gene names often overlap with common words (e.g., “MAP” and “ACE”), entity resolution is confounded by species‐specific synonyms, and phenotype associations are frequently described implicitly or across sentences [5]. Consequently, LLMs struggle to resolve gene‐phenotype relations without clearly stated contextual metadata [16, 17, 18], which is insufficient or unstructured in the source literature [7]. These factors affect model accuracy and curator agreement. Furthermore, LLMs are prone to hallucination, where fabricated but plausible‐sounding gene–phenotype relations are generated without factual support [19].

There is a lack of systematic evaluation of the use of generative AI models to extract human disease genes from the full‐text of scientific papers. One main bottleneck is the lack of high‐quality human‐curated gene lists as a ground truth. To address this issue, we used a recently published open‐access comprehensive database on human congenital heart disease (CHD) genes, called CHDgene [20]. CHD is a malformation of the heart present at birth with an incidence of 1% of live births. CHDgene is a comprehensive database containing a human‐curated list of 189 CHD‐causing genes based on human evaluation and curation of 438 publications. In this study, CHDgene serves as a gold‐standard resource for benchmarking. In domains such as CHD, there has not been any systematic benchmarking on LLM use in the field of human disease genetics. Our expertise in CHD and one of our authors’ involvement in development of CHDgene provides us with a unique opportunity to perform this benchmarking study.

We also introduced a benchmark pipeline, to evaluate LLM‐based extraction of gene–disease–phenotype associations from biomedical literature. Unlike prior approaches, our framework requires no fine‐tuning, no RAG, and no ontology integration. This framework features a multi‐dimensional evaluation schema for assessing accuracy across gene, disease, and phenotype outputs. To better capture near‐matches and address variability in biomedical terminology, we incorporated fuzzy and semantic matching into the side‐by‐side evaluation process for disease and CHD phenotype fields. The framework also includes a systematic protocol for identifying and categorizing disagreement cases—such as false positives (FPs), false negatives (FNs), and partial matches—enabling error analysis toward a more realistic and interpretable assessment of LLM performance in human genetics.

Rather than proposing a novel extraction agentic AI method or system, our main objective is to provide a reproducible benchmarking framework and empirical evidence regarding the feasibility and limitations of LLM‐assisted curation in human disease genetics.

## 2 RESULTS

### 2.1 Overall accuracies of seven one‐shot LLMs

To assess overall performance, we aggregated accuracy scores across all evaluated fields—symbol, gene name, disease association, and CHD phenotype—into a single average score per model. As shown in Figure 1, Grok‐4 achieves the highest accuracy (97.6%) across the four fields, followed by GPT‐5 (94.0%) and Qwen‐3.5 (92.9%). GPT‐4o has the lowest accuracy (87.7%), whereas Claude‐Opus‐4, Gemini‐2.5 (Pro), and DeepSeek‐R1 lie in the mid‐range.

**FIGURE 1:**
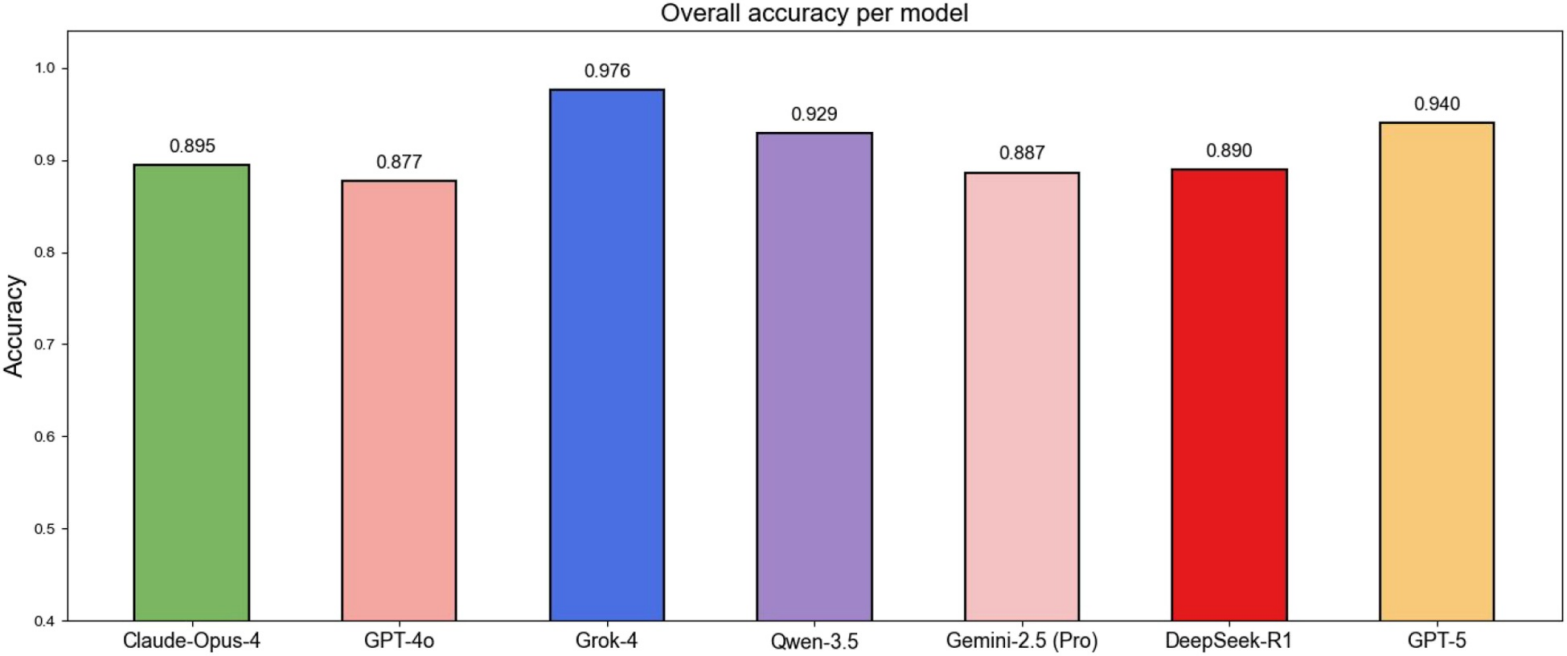
Overall accuracy of one‐shot LLMs in gene–disease–phenotype evidence synthesis. A bar plot showing the average accuracy rate (%) of seven LLMs—GPT‐4o, Claude‐Opus‐4, DeepSeek‐R1, Grok‐4, Qwen‐3.5, Gemini‐2.5 (Pro) and GPT‐5. Accuracy was measured against expert‐curated annotations from the CHDgene database. LLMs, large language models.

These findings underscore the importance of evaluating LLMs not only on isolated information types but also in terms of integrated biomedical comprehension.

### 2.2 Field‐specific performance and error profiles

To gain deeper insight, we broke down performance by each information field—gene symbol, gene name, disease, and phenotype—and by each model. Figure 2 presents this detailed accuracy breakdown. A clear trend emerged: symbols were almost uniformly extracted with higher accuracy than rest of the fields across all models. All models had near 100% success in outputting the correct gene symbol. For instance, GPT‐4o, despite being the weakest overall, still exhibited 90.2% accuracy in extraction of gene symbols.

**FIGURE 2:**
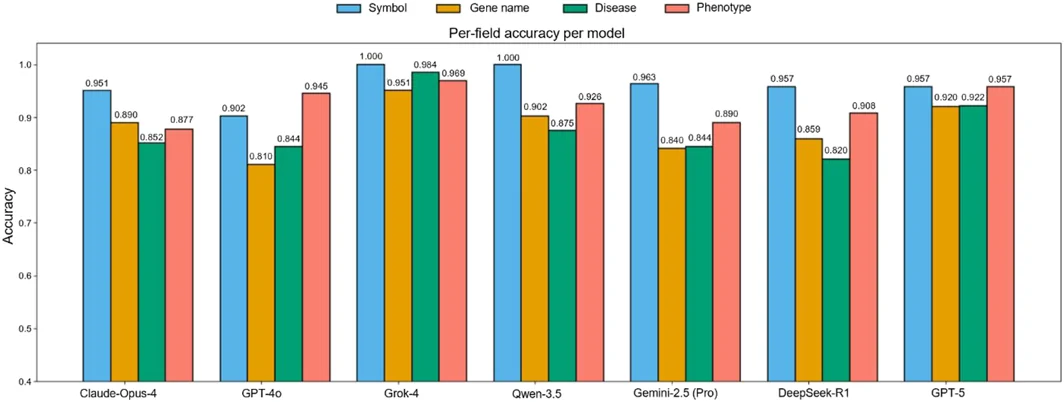
Model‐wise accuracy of gene–disease–phenotype extraction benchmarked against CHDgene gold‐standard annotations. A bar plot depicting the accuracy (%) of seven leading large language models. Accuracy was assessed across four dimensions: symbol, gene name, disease, and CHD phenotypes, based on comparison against expert‐curated CHDgene database entries. CHD, congenital heart disease.

In contrast, gene name, disease and CHD phenotype extraction were more challenging. Gene name performance: most models on average achieved 85%–95%, lowest as 81.0%; disease names performance: 85%–99% range for the majority of the models, lowest as 82.0% for DeepSeek‐R1. Phenotype field performance: top models are around 95%, Claude‐Opus‐4 is around 87.7%. But it also points to genuine difficulties: Phenotypic descriptions can be lengthy lists, sometimes buried in the text or supplementary data, and often using varied terminology.

To understand if there is any impact of lexical properties causing the field terms easier or harder for models to synthesize, we regressed the model’s wrong rate on simple and advanced lexical features for each field. We did not find any lexical features significant enough to make such a claim, suggesting lexical properties are not a primary factor in model performance for most fields. Subsequently, we turned our focus to investigate whether the errors were caused by intrinsic model biases.

A key question is how one‐shot LLM performance stacks up against prior state‐of‐the‐art results reported in similar tasks. Although our task (full‐text gene–disease–phenotype extraction) is unique, we can draw parallels to metrics in earlier works [1]: NER and RE. We approximate symbol and gene name as NER task while preserving disease and CHD phenotype extraction as relational extraction in the broader BioNLP context, we compare model‐specific F1 score in this direct benchmark (Figure 3).

**FIGURE 3:**
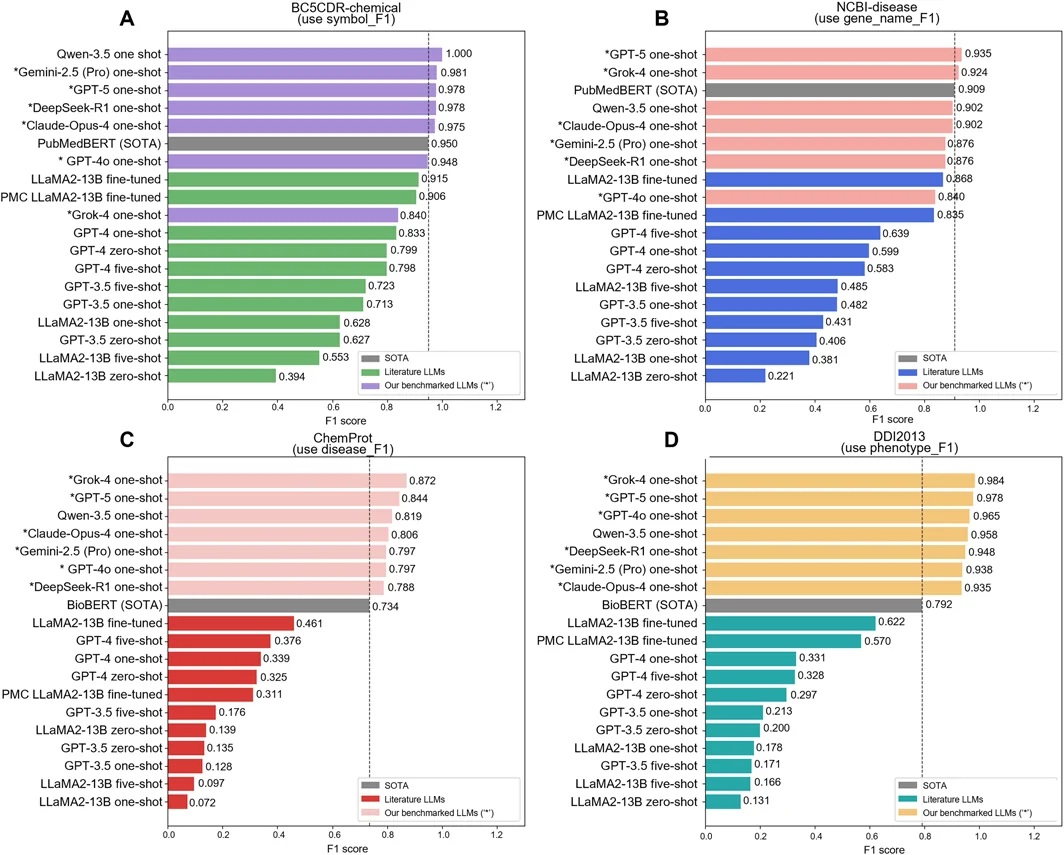
Direct benchmarking of one‐shot LLMs’ performance with current literature reported metrics. This figure compares the F1 performance of our one‐shot LLMs against traditional state‐of‐the‐art biomedical natural language processing systems and contemporary LLMs across four standard entity‐extraction benchmarks. (A) BC5CDR‐chemical, using symbol_F1. (B) NCBI‐disease, using gene_name_F1. (C) ChemProt, using disease_F1. (D) DDI2013, using phenotype_F1. LLM, large language model; NCBI, National Center for Biotechnology Information.

Figure 3A (BC5CDR‐chemical, using symbol_F1): Our one‐shot prompted LLMs achieve near‐perfect performance, with Qwen‐3.5 one‐shot, Gemini‐2.5 (Pro) one‐shot, and GPT‐5 one‐shot all matching or surpassing PubMedBERT (state‐of‐the‐art [SOTA]), outperforming all GPT‐4, GPT‐3.5, and LLaMA2 variants across one‐shot, few‐shot, and zero‐shot settings reported in the literature.

Figure 3B (National Center for Biotechnology Information disease, using gene_name_F1): Our one‐shot prompted LLMs again ranks among the top performers, outperforming PubMed BERT and all instruction‐tuned LLMs, with GPT‐5 leading.

Figure 3C (ChemProt, using disease_F1): Relation‐style disease extraction remains challenging for many LLMs; nevertheless, Grok‐4 one‐shot, GPT‐5 one‐shot, and Qwen‐3.5 one‐shot achieve the highest scores, substantially exceeding BioBERT SOTA and greatly outperforming GPT‐4 and GPT‐3.5 in all prompting modes.

Figure 3D (DDI2013, using phenotype_F1): Our one‐shot prompted LLMs achieves strong phenotype‐extraction performance, with Grok‐4 one‐shot, GPT‐5 one‐shot, and GPT‐4o one‐shot at the top of the benchmark. Traditional SOTA (BioBERT) and surface LLaMA2 baselines perform substantially worse in comparison.

In summary, our one‐shot prompted LLMs’ results compare favorably with, and in some aspects exceed, previously reported metrics in related literature. We achieve near‐perfect extraction accuracy in a targeted domain, without special model tuning, whereas prior benchmarks on analogous tasks showed lower accuracy.

### 2.3 Prompt‐specific gate‐keeping effectiveness

The one‐shot prompt we used does more than ask the model to list a gene, disease and CHD phenotype; it also lays out a set of gate‐keeping rules that must be satisfied before any extraction is attempted. If the provided paper’s PDF file does not contain any human genetic content. Specifically:

- No gene symbols or gene full names are mentioned.
- No references are made to genetic diseases, including CHD.
- There is a complete absence of gene–disease–phenotype relationships or any molecular biology terminology relevant to CHDgene extraction.

As such, the prompt correctly fails this document because it lacks any extractable content within the scope of human genetics or CHD. Our one‐shot prompt successfully distinguishes genetics papers from the 406 non‐genetics articles by matching against human genetics signatures with 100% gate‐keeping effectiveness, an example output generally involving sentences such as “The provided paper does not discuss any genes or genetic research and does not include information on gene symbols, gene names, or associated diseases/phenotypes. Therefore, I cannot produce the requested format based on this document.” True genetics papers repeatedly reference symbols, gene names, disease, and CHD phenotypes, excluded papers lacking these markers or mentions genes only parenthetically as background.

## 3 DISCUSSION

Our benchmark showed that several LLMs (notably Grok‐4, GPT‐5, etc.) can achieve accuracy above 90%, essentially approaching the reliability of manual extraction for many entries. This presents as the first systematic validation of LLMs for this specific task in full‐text human genetics against expert‐curated databases. These observations echo broader concerns about LLM output veracity in biomedical contexts [1]. By analyzing errors in depth, we identified common failure modes (e.g., over‐predicting very common defects or struggling with unusual disease descriptions), which provide actionable targets for refinement.

### 3.1 Fully automatic evidence synthesis remains a challenge

While our results are encouraging, we underscore that we evaluated models in a one‐shot setting on known gene‐associated papers. This is a necessary first step before deploying LLMs to find new or rare gene–disease relationships. In a real curation workflow, one might task an LLM with scanning any new paper for gene–disease–phenotype claims. Our gate‐keeping experiment suggests LLMs can correctly ignore irrelevant papers (100% specificity in our test). Our error analysis indicates that some phenotypic details were missed even in known cases. Thus, a fully automated pipeline might still miss novel or rare findings if they are described in atypical ways. This aligns with the observation that GPT‐4 alone achieved low accuracy in identifying the causal gene for rare disease cases [18]. In terms of use case (populating databases from literature), a practical scenario could be an AI‐assisted curator: the LLM proposes a structured entry from a paper, and a human curator verifies and fills any gaps.

### 3.2 Benchmarking with prior literature

In comparing our results to prior studies, we see some common issues. Hallucinations, inconsistencies, and missing information are recurrent themes when LLMs are applied to any factual task. We showed that providing even one example (one‐shot) in the prompt and explicit instructions greatly mitigated these issues for our task, supporting the recommendation that advanced prompt engineering or one‐shot examples can significantly improve biomedical LLM performance [1].

It is noteworthy that some prior works integrated retrieval augmentation or fine‐tuning [4] to boost performance. Those approaches can improve precision, for example, RAG‐human phenotype ontology (HPO) virtually eliminated hallucinated HPO terms by forcing choices from a database [21]. In our case, we essentially relied on the model’s internal knowledge of CHD terminology, which was adequate. A hybrid approach could involve feeding the LLM a list of allowed phenotype terms (from an ontology) to choose from. This might prevent non‐existent terms or too‐general terms from creeping in. The risk is it might make the model too rigid and miss variants. A middle ground is ontology‐constrained decoding, something others have suggested as well. For instance, if the model is about to output a phenotype, one could post‐process to map it to the closest HPO term, thus standardizing outputs [22].

### 3.3 Toward reliable model‐assisted curation

Based on our extended analyses, we propose a few strategies to further improve reliability:

• Ontology anchoring: As mentioned, linking outputs to controlled vocabularies (online Mendelian inheritance in man for diseases, the HPO for phenotypes) can reduce synonym mismatches and prevent non‐standard terms. For example, if a model says “hole in the heart,” mapping it to “atrial septal defect (HP:0001631)” provides clarity.

• Confidence‐based gating: Recent studies [23] suggest using the model’s own logits or an ensemble to gauge uncertainty. In curation, we could have the model flag any low‐confidence extraction for human review. This could effectively catch those cases where the model was likely guessing.

• Prompt constraints for specificity: We can refine the prompt to avoid “umbrella terms.” For instance, instruct the model: “Prefer specific defect names over general terms.” We noticed models sometimes output a broad term like “CHD” as a phenotype, which is not useful. Some prompt experiments where we explicitly forbade certain generic phrases did push models to list specifics only. This kind of rule‐based prompt tweak is quick and does not require training.

• Multi‐model agreement: As noted, requiring consensus among multiple LLMs could be a powerful filter. If GPT‐5, Grok‐4, and Claude‐Opus‐4 all independently extract “ventricular septal defect (VSD),” it’s very likely correct. If one says “VSD” and another doesn’t mention it, which might need a closer look. Such cross‐checking can be automated.

### 3.4 Limitations and future directions

Several limitations warrant consideration. The benchmark corpus remains limited to English‐language papers, and multilingual evaluation is needed to ensure global applicability. All analyses were performed under one‐shot prompting; future comparisons with few‐shot, chain‐of‐thought, or RAG‐augmented prompting [19] may yield additional insights. Our schema captured only four core fields; future work could incorporate variant‐level annotations, inheritance patterns, or population‐specific genetic effects.

Several limitations of our study should be acknowledged. First, our benchmark is limited to English‐language papers on CHD. Genetic literature in other languages or on other disease domains might pose new challenges (for instance, terminology differences). Extending evaluation to non‐English or multilingual corpora is an important next step to ensure global utility. Second, we used only the one‐shot prompting. It would be informative to compare chain‐of‐thought prompting. Perhaps having a model reason stepwise could reduce hallucinations. We plan to explore these variants in future work. Third, our schema captured only four fields. Real databases might want richer information: genetic variant details, mode of inheritance, population studied, etc. Incorporating such additional fields is non‐trivial—some might be even harder for LLMs (e.g., parsing a specific variant ID from text). But our framework could be extended by adding those to the prompt and scoring accordingly. It may require more complex instructions and examples.

Another limitation is that our evaluation treated CHDgene’s current content as ground truth. If a paper actually had a phenotype not recorded in CHDgene, our scoring would mark it as a false positive (because it is “not in CHDgene”). We did come across a couple of instances where the LLM output a reasonable phenotype that a curator might have omitted in CHDgene. In the future, as LLMs scour literature, they might highlight genuine novel findings, which is a positive outcome, but our evaluation framework would initially view it as an “error.” Distinguishing model hallucination from legitimate new evidence is a challenging aspect; one approach is to have a secondary verification step, perhaps using the LLM to cite the exact sentence for each output field as justification.

In addition, we report gene‐level accuracy by aggregating extractions across all supporting papers for each gene, in order to align with CHDgene’s database granularity. This aggregation increases sensitivity to at least one supporting extraction per gene but can mask document‐level variability: a single correct extraction from multiple papers suffices for a gene‐level match. Therefore gene‐level accuracy should not be interpreted as guaranteed per‐document reliability; document‐level metrics and curator verification are recommended for deployment.

Finally, although we tested seven models, the landscape of LLMs is evolving rapidly. New models could alter the relative performance. We observed GPT‐4o lagged behind some newer entrants such as Grok‐4, which suggests continuous improvements are being made by model providers. An open question is how much of our success is due to the prompt/framework versus the inherent model power. This baseline could likely be raised by techniques we discussed, or by fine‐tuning an open model on a small set of examples (as a last resort). For now, using top‐tier models seems to pay off substantially for difficult extraction tasks.

## 4 CONCLUSION

We presented a systematic benchmark of seven LLMs for automated extraction of gene–disease–phenotype relationships from full‐text scientific literature. Without specialized training, our best one‐shot prompted model achieved a gene‐level match accuracy of 97.6% on a focused genetic disease domain, outperforming state‐of‐the‐art models under this aggregate metric.

Notably, our benchmark is limited to CHD, a relatively well‐studied set of genes with established phenotypic associations. Performance on less‐studied diseases, those with complex inheritance patterns, or those with highly variable nomenclature may differ substantially. Therefore, our conclusions should be interpreted as evidence of feasibility and a benchmark for CHD specifically; generalization to other diseases requires further validation. We have made our evaluation framework available to facilitate such extensions.

However, it is important to note that this evaluation allowed a single correct extraction across multiple documents to count as a gene‐level success; consequently, per‐document reliability may be lower than this. A single correct extraction from multiple papers suffices for a gene‐level match, which may overestimate per‐document model reliability. Hence gene‐level accuracy should not be interpreted as guaranteed per‐document reliability. Owing to the fact that our aggregation strategy aligns with the CHDgene database’s gene‐level representation, it sacrifices document‐level resolution. Future work should develop and report document‐level metrics (e.g., extraction per paper) to better characterize model accuracy across individual articles.

Through comprehensive evaluation and error analysis, we showed that modern LLMs can be reliably prompted to output structured biomedical information, given appropriate guidance and validation. Our prompt’s design and results set it apart as an advance in biomedical text mining: it achieves high accuracy without fine‐tuning or external resources, in contrast to previous systems that required extensive training data or complex pipelines. By explicitly comparing with recent literature, we also illustrated how one‐shot prompted LLMs, while being simpler and more immediately deployable as well as widely accessible, yet yields competitive results in the domain.

## 5 MATERIALS AND METHODS

### 5.1 Dataset construction and retrieval

To assess the ability of LLMs to extract gene–disease–phenotype information for CHD in controlled comparison, we leverage the CHDgene repository [20], a high‐quality manually curated database. CHDgene integrates published findings of gene‐level associations with congenital heart defects and serves as our benchmark for genetic evidence synthesis. We restricted our analysis to protein‐coding genes listed in CHDgene, each associated with at least one PubMed‐indexed primary research article included under Selected Reference section in CHDgene database. Consistency level of information extracted by seven state‐of‐the‐art, off‐the‐shelf, and commercially available LLMs—Qwen‐3.5, Gemini‐2.5 (Pro), Grok‐4, GPT‐5, GPT‐4o, Claude‐Opus‐4, and DeepSeek‐R1—are quantified across multiple dimensions including gene symbol, gene name, disease associated, and CHD phenotypes.

We collected 189 CHD genes from the CHDgene’s gene list, and 180 genes have one or more full‐text articles as supporting evidence, the rest of the 9 genes were filtered out due to lack of full‐text literature. We have compiled custom web scraping scripts in Python (v3.12) and retrieved the following items (Figure 4):

- The gene symbol and full name
- Disease annotations (e.g., “Heterotaxy”, “TOF”)
- Associated CHD phenotypes
- Indexed biomedical literature with PubMed links

**FIGURE 4:**
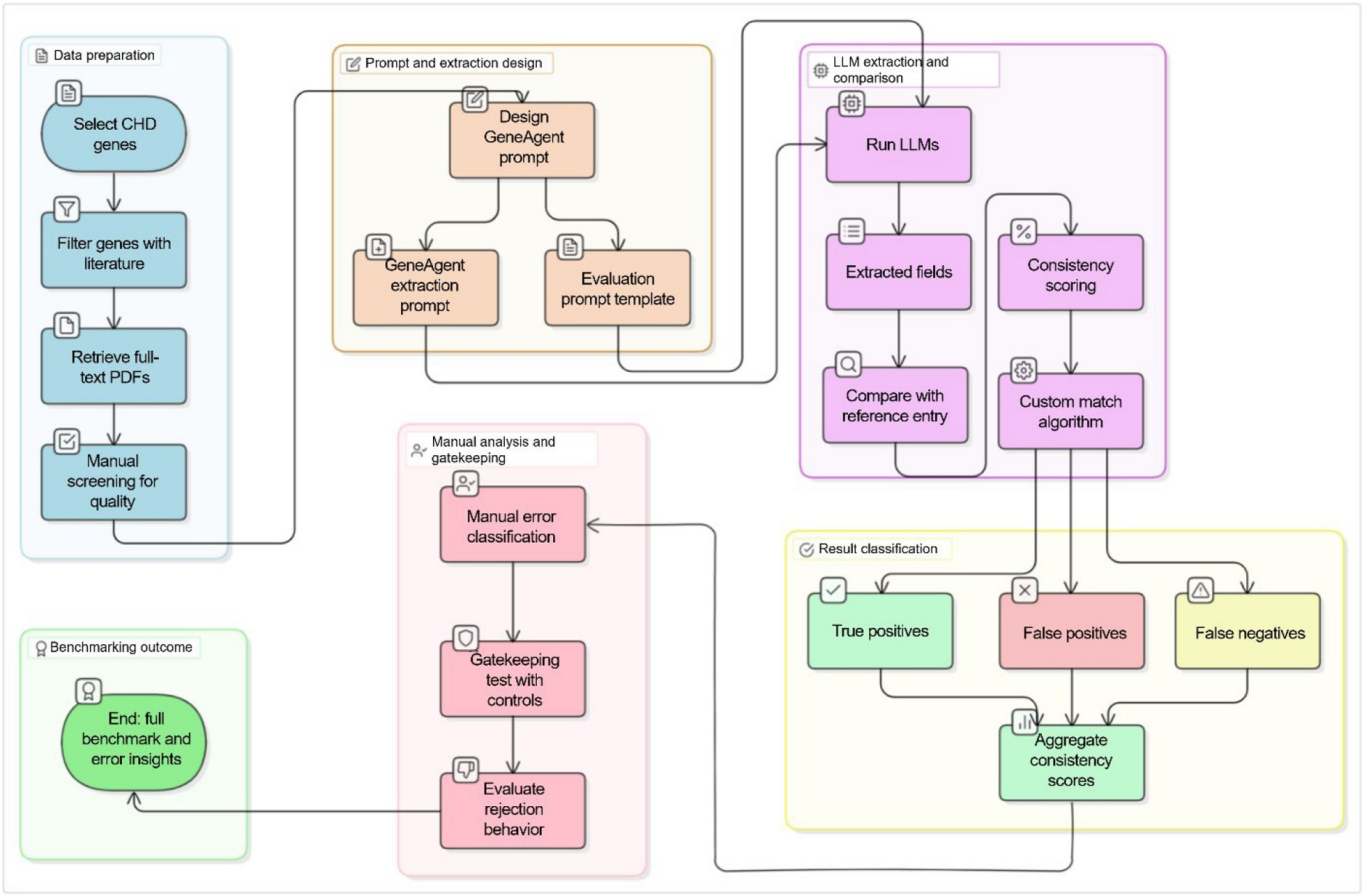
Overall pipeline of the benchmark study design. An overview of pipeline for benchmarking LLM‐based evidence synthesis of gene–disease–phenotype relationships. The workflow includes data preparation (blue), prompt design (orange), LLM information extraction framework (purple) with result evaluation (yellow), manual error analysis (pink), and final benchmarking (green). LLM, large language model.

The data are all manually verified for correctness. This benchmark data set contains 180 unique CHD‐associated genes, containing a total of 406 scientific papers. This corpus formed the benchmark for evaluating the accuracy of LLM‐based extraction.

To construct the test set for LLM evaluation, we retrieved full‐text PDFs of the selected literatures from PubMed Central. PDF files were stored and labeled with standardized filenames containing the gene symbol. Manual inspection was performed to ensure PDF quality and relevance by humans. Articles not containing original genetic data or not in English were excluded, ensuring 406 high‐quality PDF documents as the final test corpus.

### 5.2 Prompt design

We developed a one‐shot prompt (Figure 5) which is designed to emulate the structure of entries in CHDgene. The goal was to instruct the LLM to extract, in a structured and faithful manner, the gene symbol, full gene name, associated CHD phenotype(s), and disease term (if specified) from the full‐text scientific paper.

**FIGURE 5:**
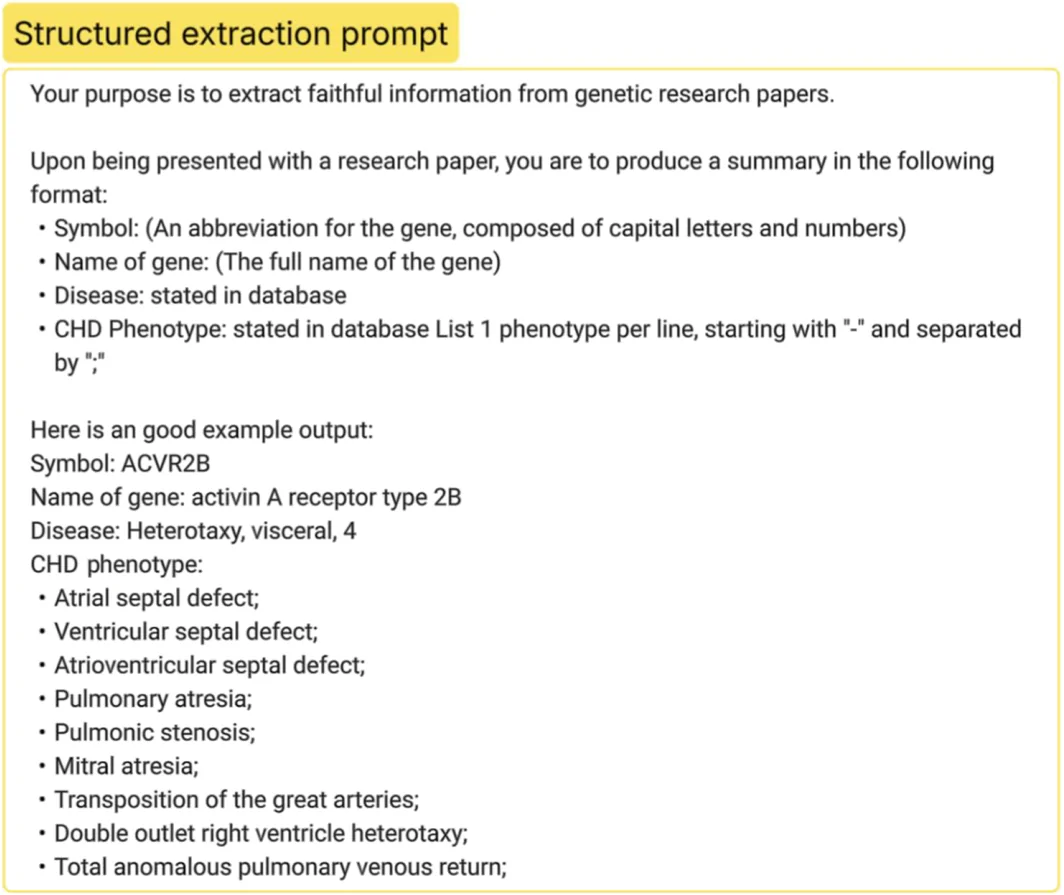
One‐shot extraction prompt used in benchmark study. A standardized one‐shot extraction prompt was used to generate structured gene–disease–phenotype outputs from full‐text articles. Extracted outputs were subsequently evaluated against CHDgene reference records using a structured accuracy scoring protocol with multi‐dimensional assessment across gene, disease, and phenotype fields.

The prompt contains the following components:

- A concise instruction (system message) defining the task of structured evidence synthesis.
- An example with a known CHDgene record (e.g., “Symbol: ACVR2B; Name of gene: activin A receptor type 2B; Disease: Heterotaxy, visceral, 4; CHD phenotype: Atrial septal defect; Pulmonary atresia; …”)

No retrieval augmentation, external tools, or task‐specific fine‐tuning were applied. All models were applied with the same prompt, no model‐specific modification on prompt is involved.

### 5.3 Evaluation metrics

To systematically evaluate the evidence synthesis performance of LLMs, we assessed the structured outputs along multiple dimensions aligned with the CHDgene database schema. For each input paper, the LLM‐generated evidence was compared to the gold‐standard CHDgene record and scored using a multi‐dimensional accuracy framework.

### 5.4 Scoring system: Performance evaluation

#### 5.4.1 Gene‐level scoring criteria

Each synthesized field—gene symbol, gene name, disease, and phenotype list—was recorded independently for every model output against the CHDgene database entry. The raw records are 406, each record refers to one literature associated per gene.

To evaluate overall performance for each gene, we aggregated all model output across all records, yielding 180 clean records. For each gene, we compiled a unified, deduplicated list of synthesized outputs for each field. We report gene‐level accuracy by aggregating extracted output across all supporting papers for each gene, in order to align with CHDgene’s database granularity.

The final, gene‐level score was determined by a binary comparison to the ground truth: An item was counted if at least one correct output for that field appeared in the aggregated list. If no correct output was present, it was not counted.

We employed a binary scoring system:

- Match (1): At least one entity from aggregated LLM output results exactly matched or was semantically fuzzily matched to the CHDgene entry’s ground truth.
- No match (0): The aggregated results do not contain a single correct output present in the CHDgene (e.g., an incorrect gene name, an unrelated disease, or unverifiable information).

For each model, the per‐field accuracy is calculated through the count of matches divided by a total of 180 genes, and the overall accuracy is by taking an average of the per‐field accuracies of all fields.

#### 5.4.2 Direct comparison with prior literature

To further assess models performance against other state‐of‐the‐art models studied in previous literature, we summarize the number of true positives, FPs, and FNs used to calculate three key metrics: precision, recall, and the F1 score (Table 1). F1 scores were used in determining the performance of the model for the direct benchmarking purpose.

**TABLE 1:** F1 score calculation formula.

Measure	Definition	Formula
True positives (TPs)	Responses containing CHD existing phenotypes supported by the CHDgene entry and the PDF content	‐
False positives (FPs)	Responses containing CHD phenotypes unsupported by either the CHDgene entry or the PDF content	‐
False negatives (FNs)	Cases where the reference paper clearly contained CHD phenotype information, but the LLM failed to return any structured output	‐
Precision	Precision measures the proportion of correctly extracted phenotypic terms among all the terms predicted by the tool	$P\text{recision}=\frac{\text{TP}}{\text{TP}+\text{FP}}$
Recall	Recall measures the tool’s ability to retrieve all the phenotypic terms that should have been extracted, considering the true terms in the document	$\text{Recall}=\frac{\text{TP}}{\text{TP}+\text{FN}}$
F1 score	The F1 score combines precision and recall into a single metric, giving a better overall measure of a tool’s performance in phenotype extraction. Measures how well the model extracts the phenotypic terms from literature	$F1\;\text{score}=2\times \frac{\text{Precision}\times \text{Recall}}{\text{Precision}+\text{Recall}}$

Abbreviation: CHD, congenital heart disease.

### 5.5 Benchmark design for LLM accuracy evaluation

#### 5.5.1 Original preprocessing and exact match

We initially established an exact‐match scoring strategy, where perfect alignment of extracted terms with the CHDgene database entries was required for positive scoring. However, recognizing inherent variations in biomedical nomenclature, we progressively enhanced our matching criteria. Phenotype terms were only standardized by lowercasing, punctuation removal, and singular/plural harmonization (e.g., “defects” unified to “defect”) and these fields were evaluated using exact match.

#### 5.5.2 Pre‐normalization and enhanced matching strategies

With enhanced matching, we evaluate extracted fields against CHDgene using deterministic rules (Table 2). For case‐insensitive comparisons we apply .normalize(), .strip() and .lower(). Symbol is case‐insensitive equality; Gene name and disease use approximate matching; CHD phenotypes use set overlap. Fuzzy match uses Python package difflib function SequenceMatcher(None, a, b).ratio() with a fixed ratio or threshold of 0.85 for the field; We split on semicolon (;) and declare a match if there is any exact element‐level overlap. Missingness is explicit: both empty → match; exactly one empty → non‐match. For phenotypes, we compute *t*_p_, *f*_p_, *f*_n_, precision, recall, and F1 from set operations and also report a binary indicator defined as “any match.” To obtain one record per gene per model, we group by gene_id and evaluate the first occurrence per group (file order). The pipeline uses only the following Python packages: difflib, re, numpy, pandas, and openpyxl.

**TABLE 2:** Matching strategies adopted in evaluating the performance of large language model extracted fields against CHDgene entries.

Field	Matching strategies
Symbol	Case‐insensitive normalized exact match
Name of gene	Case‐insensitive normalized fuzzy match (see fuzzy_match function in aggregate_result_per_gene.ipynb)
Disease	Case‐insensitive normalized fuzzy match (see fuzzy_match function in aggregate_result_per_gene.ipynb)
CHD phenotypes	Case‐insensitive normalized fuzzy match (see fuzzy_match function in aggregate_result_per_gene.ipynb)

Abbreviation: CHD, congenital heart disease.

The threshold of 0.85 was selected empirically based on a manual review of mismatched examples. This threshold balanced the acceptance of unstandardized or unnormalized terms (e.g., “zinc finger MYND‐type containing 8” vs. “zinc finger MYND domain‐containing protein 8”) while rejecting biologically incorrect terms that appeared semantically similar (e.g., “spalt like transcription factor 4” vs. “spalt like transcription factor”).

#### 5.5.3 Negative controls

To ensure robustness, we tested LLM behavior on some negative control papers, which consists of 406 randomly sampled articles from PubMed Central that are not related to CHD or genetics. These samples were selected such that the correct response when presented to the system is that the system will indicate that the paper does not contain any CHDgene related information for extraction.

## AUTHOR CONTRIBUTIONS

**Danqing Yin**: Formal analysis; methodology. **Matthew Ka Siu Leung**: Conceptualization; data curation; formal analysis; investigation. **Darren Wan Ho Pun**: Conceptualization; data curation; formal analysis; investigation; methodology. **Fiona Haixin Chen**: Conceptualization; data curation; formal analysis; investigation; methodology. **Julie Yujin Kwon**: Conceptualization; data curation; formal analysis; investigation; methodology. **Xinyi Lin**: Conceptualization; data curation; formal analysis; investigation; methodology. **Joshua W. K. Ho**: Supervision.

## CONFLICT OF INTEREST STATEMENT

The authors declare no conflicts of interest.

## ETHICS STATEMENT

This study did not involve human participants, animal subjects, or clinical trials. All experiments and manual literature screenings were conducted using previously published, publicly available. Therefore, ethical approval from an institutional review board (IRB) or an independent ethics committee was not required.

## Data Availability

All processing scripts were implemented in Python (v3.12). Our entire pipeline, including prompt templates, web scraping scripts, results evaluation notebooks, and results, is made publicly available at GitHub webset (holab‐hku/CHDgeneBench). The benchmark datasets are available upon request.
